# Fatty acids, inflammation and intestinal health in pigs

**DOI:** 10.1186/s40104-015-0040-1

**Published:** 2015-09-09

**Authors:** Yulan Liu

**Affiliations:** Hubei Collaborative Innovation Center for Animal Nutrition and Feed Safety, Hubei Key Laboratory of Animal Nutrition and Feed Science, Wuhan Polytechnic University, Wuhan, 430023 China

**Keywords:** Fatty acids, Inflammation, Intestine, Pigs

## Abstract

The intestine is not only critical for nutrient digestion and absorption, but also is the largest immune organ in the body. However, in pig production, inflammation induced by numerous factors, such as pathogen infection and stresses (e.g., weaning), results in intestinal mucosal injury and dysfunction, and consequently results in poor growth of pigs. Dietary fatty acids not only play critical roles in energy homeostasis and cellular membrane composition, but also exert potent effects on intestinal development, immune function, and inflammatory response. Recent studies support potential therapeutic roles for specific fatty acids (short chain and medium chain fatty acids and long chain polyunsaturated fatty acids) in intestinal inflammation of pigs. Results of these new lines of work indicate trophic and cytoprotective effects of fatty acids on intestinal integrity in pigs. In this article, we review the effect of inflammation on intestinal structure and function, and the role of specific fatty acids on intestinal health of pigs, especially under inflammatory conditions.

## Introduction

In developing management and nutritional strategies to maximize growth performance and health of pigs, it is critical to consider the effect of inflammation on gastrointestinal (GI) function. As we know, the GI tract is not only an important organ for digestion, absorption and metabolism of dietary nutrients, but also is the largest immune organ in the body, which comprises more than 70 % of the body’s immune cells [1]. However, in pig production, the pigs encounter numerous pathogenic and nonpathogenic challenges, which results in activation of GI immune system. Activation of the GI immune system leads to the production of a diverse set of specialized cells and signaling molecules, especially pro-inflammatory cytokines such as tumor necrosis factor (TNF)-α, interleukin (IL)-1β and IL-6 [2, 3]. Over-production of these cytokines results in intestinal mucosal injury and dysfunction, and consequently result in poor growth of pigs [2]. Consequently, pigs suffered from intestinal infections (such as enterotoxigenic *Escherichia coli* infection) have lower feed intake, weight gain and gain/feed ratio than the control pigs [4, 5]. Although it is important that the GI immune system be activated to deal with invading pathogens in cases of high risk or confirmed pathogen exposure, nutritional strategies that avoid excessive activation of GI immune system are important means to improve the efficiency of pig production.

Fatty acids are a major energy source, important components of the cell membrane, metabolic substrates in many biochemical pathways, cell-signaling molecules, and play a critical role as immune modulators [6–8]. Research has shown that fatty acids, especially n-3 polyunsaturated fatty acids (PUFA), exert beneficial effects on inflammatory bowel diseases in animal models and clinical trials [6, 7]. The protective role of these fatty acids in the intestine is closely related to their inhibitory effects on the over-release of intestinal inflammatory mediators, especially pro-inflammatory cytokines [6–8]. Recently, the studies in pig nutrition also support potential therapeutic roles for the specific fatty acid [short chain and medium chain fatty acids, and long chain PUFA including n-3 PUFA, arachidonic acid (ARA) and conjugated linoleic acids (CLA)] in intestinal inflammation [9–11]. In this article, we mainly focus on the effect of inflammation on GI structure and function, and the role of specific fatty acids on intestinal health of pigs, especially under inflammatory conditions.

## Inflammation and gastrointestinal function

Inflammation is a fundamental aspect when considering the functioning of the GI tract. A healthy GI tract is thought to be in a constant state of “controlled” inflammation as a result of the proximity of a dense population of bacteria in the GI lumen, dietary antigens, and toxins. It is often thought that the intestine of a conventional, clinically healthy pig is not inflamed. However, in fact, when compared with a germfree pig, the intestine of a conventional pig displays markedly up-regulated expression of pro-inflammatory cytokines, infiltration of immune cells, and organization of lymphoid follicles and Peyer’s patches [12]. Thus, GI immune system activation associated with a “normal” commensal microbiota has significant effects on intestinal morphology and the ability to digest and absorb nutrients of the pigs. When overt intestinal infections (such as enterotoxigenic *Escherichia coli* and *Salmonella typhimurium* infections) occur, inflammatory responses are drastically amplified, and intestinal morphology and function are further impaired [13–15]. In addition, inflammation induced by stresses such as weaning has also a substantial impacts on intestine [3]. In the next section, we examine the effects of inflammation on intestinal morphology, digestive and absorptive abilities and barrier function.

### Morphology

There is clear evidence in the literature that inflammation induced by several factors causes drastic morphologic changes to the pig intestine. Colonization of germ-free piglets with a normal flora resulted in two-fold decrease of villus height and deepening of the crypt compared to germ-free pigs. Shorter villi in colonized pigs relative to germfree pigs indicates a substantial contribution of commensal bacteria to apoptosis and sloughing of enterocytes [12]. Oral infection with *Escherichia coli* K88^+^ decreased villus height, villus height:crypt depth ratio, villus area, and villus volume compared with the control pigs. However, the crypt depth was not affected [4]. Alterations in intestinal morphology are not restricted to inflammation in the intestine. In a model of intraperitoneal LPS-induced sepsis, various intestinal morphologic changes, such as villus atrophy, submucosal edema, epithelial vacuolation, frank hemorrhage and necrosis have been observed in pigs [2, 16–18]. Moreover, as we know well, the inflammation associated with stresses such as weaning also have deleterious effects on the intestinal morphology of pigs. For example, Hu et al. [19] found that pro-inflammatory cytokines such as IL-6 and TNF-α mRNA levels increased considerably and coincided with a significant decrease in villus height and a significant increase of crypt depth on days 3 and 7 post weaning of piglets. Inflammation may induce intestinal morphologic changes directly or indirectly (mediated by reduced feed intake). Inflammation can result in reduction of feed intake in pigs [16]. The feed intake reduction alone also impairs pig intestinal morphology [20, 21]. Therefore, the effects of inflammation on intestinal morphology may be confounded and exacerbated by the dramatic reduction in feed intake.

### Digestive and absorptive function

The alterations in intestinal morphology associated with inflammation can have consequences for intestinal mucosa functions, including nutrient digestion and absorption. Willing and Van Kessel [22] reported that colonization of neonatal gnotobiotic pig with a normal flora led to the reduced activity of brush-border enzymes, including lactase phloryzin hydrolase (LPH) and aminopeptidase N (APN). The host compensated for reduced activity of APN by increasing its gene expression, however, it was unable to return enzyme activity level to that of a germfree pig. In addition, Trebichavsky et al. [23] demonstrated that orally infection with virulent LT2 strain of *Salmonella enterica* serotype Typhimurium caused a significant decrease of gamma-glutamyl transpeptidase (GGT) activity in both the jejunum and ileum. Infection with the rough mutant of *Salmonella enterica* serotype Typhimurium caused a decrease of GGT activity only in the ileum. However, the activities of other brush border enzymes including lactase, sucrase, glucoamylase, alkaline phosphatase and dipeptidylpeptidase IV were not affected significantly after infection. In addition to enteric infection, systemic inflammation induced by intraperitoneal LPS challenge also results in decreased intestinal disaccharidase activities in jejunum and ileum of weaned pigs [17, 18]. Moreover, inflammation related to weaning results in a drastic decrease in sucrase and lactase activities of small intestine between days 0 and 2 post weaning of piglets [3].

### Gut barrier function

In addition to digestive and absorptive function, inflammation can have a detrimental effect on intestinal barrier function. Intestinal barrier function is commonly described as the capacity of the GI epithelium to prevent the penetration by luminal bacteria and dietary allergens into the mucosa [1]. Several components form the multi-layered intestinal barrier. In the lumen, gastric acid and pancreatic juice degrade bacteria and antigens. In addition, commensal bacteria inhibit pathogen colonization by producing antimicrobial substances [24]. Moreover, the microclimate close to the epithelium is composed of the unstirred water layer, glycocalyx and mucus layer, which prevents bacterial adhesion and contains antimicrobial products secreted by Paneth cells and secretory IgA from the enterocytes [25]. Below the unstirred water layer, glycocalyx, and mucus layer, there are epithelial cells separated by junctions that represent homo- and heterotypic binding of extracellular domains of tight junction proteins [24]. The intestinal epithelium is constructed of a monolayer of epithelial cells including columnar epithelial cells, Paneth cells, goblet cells, and M cells. These cells cover the mucosa and play a central role in intestinal mucosal barrier and host immune response [26]. Of them, the Paneth cells synthesize and secrete antimicrobial peptides such as lysozyme and defensins. These peptides have antimicrobial activity against a number of potential pathogens. The goblet cells secrete mucus. The mucus has antimicrobial role and forms a highly charged gel that acts as a physical barrier. These molecules limit bacterial access to the epithelial surface [26]. Directly below the epithelium, numerous innate and acquired immune cells also play a critical role in regulation of mucosal barrier and host immune response [26]. Among these immune cells, innate lymphoid cells and mast cells are key players, which have multiple roles in maintaining intestinal homeostasis and innate immune surveillance to protect the host against invading enteric pathogens, and which also act as gatekeepers to the mucosal compartment [27].

Inflammation has a marked effect on barrier permeability [1]. Several pathogens have been demonstrated to directly impair intestinal tight junctions either through membrane adhesion or secreted toxins, resulting in cell damage and apoptosis or through destabilization of tight junction protein complexes. For example, Muza-Moons et al. [28] reported that infection of intestinal epithelial cells with enteropathogenic *Escherichia coli* resulted in impaired barrier function and aberrant tight-junctional protein complexes. In addition, systemic inflammation induced by inflammatory stimuli such as a single injection of LPS leads to injury of intestinal barrier function and down-regulated expression of occludin and claudin-1 in weaning pigs [2]. However, Rakhshandeh et al. [29] reported that repeated LPS injection did not alter transepithelial resistance in the ileum of growing pigs although it decreased nutrient digestibility and increased active glucose transport. The reasons for this discrepancy might be that the pigs develop a tolerance to the multiple, subsequent LPS challenges [30], and/or the growing pigs are more resistant to LPS challenge relative to weaning pigs. Moreover, early weaning also results in increased intestinal permeability and decreased expression of tight junction proteins in piglets [19]. Furthermore, early weaning impairs innate mucosal immune responses to enterotoxigenic *Escherichia coli* challenge [31]. During inflammation, pro-inflammatory cytokines play a critical role in impairment of intestinal barrier function. Of them, TNF-α and interferon γ (IFNγ) are the primary regulators of tight junctions. These cytokines have been demonstrated to directly decrease occludin expression [32], reorganize tight junction proteins, and impair barrier function [33].

## The impact of fatty acids on intestinal health of pigs

**Fig. 1:**
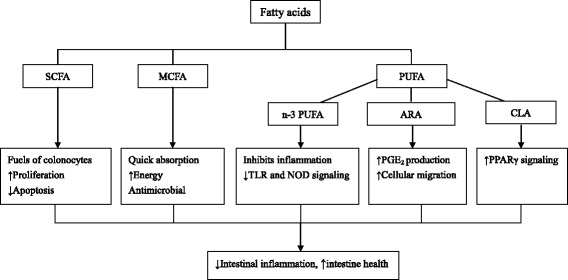
Mechanism by which fatty acids might exert benefical effects on intestinal heath in pigs. ARA: arachidonic acid; CLA: conjugated linoleic acid; MCFA: medium-chain fatty acid; NOD: nucleotide binding oligomerization domain protein; PGE_2_: prostaglandin E_2_; PPARγ: proliferator-activated receptor-γ; PUFA: polyunsaturated fatty acid; SCFA: short-chain fatty acid; TLR: toll-like receptor

According to the above-mentioned viewpoints, we might reduce inflammation-induced gut dysfunction by several means, such as removal of all inflammatory stimuli, depression of intestinal immune response, and use of pro-inflammatory cytokine antagonists to alleviate the negative effect of these cytokines [34]. However, these means are neither viable nor desirable options in commercial pig production system [34]. There are dietary strategies that reduce intestinal damage that results from inflammation or limit induction of inflammatory pathways. In this section, we consider the roles of several specific fatty acids as a means to improve intestinal health or limit intestinal inflammation (Table 1 and Fig. 1).

**Table 1:** Summary of studies investigating the effect of fatty acids on intestinal health of pigs

Fatty acids	Effects	Animals	References
SCFA	↓Intestinal atrophy, ↑structural indices of GI adaptation, ↑enterocyte proliferation, ↓enterocyte apoptosis	TPN-fed neonatal pigs	[40]
	↓Diarrhoea incidence, ↑serum IgG concentration and jejunal IgA^+^ cell count	Weaned piglets	[42]
	↑ Intestinal morphology and disaccharidase activity	Newly weaned piglets	[43]
	↓Intestinal injury by ↓ apoptosis, ↑ tight-junction formation, ↑EGFR signaling	Pig model of acetic acid-induced colitis	[44]
	↑Recovering of intestinal wound healing	Porcine IPEC J2	[45]
	↑Intestinal morphology, ↓total viable counts of proximal colon *Clostridium* and *Escherichia coli*, ↓serum TNF-α and IL-6 levels, and intestinal DNA-binding activity of NF-κB	Weaned piglets	[46]
	↓Gastric emptying and intestinal mucosa weight	Piglets before or after weaning	[47]
	↑Host defense peptide gene expression	IPEC-J2	[48]
MCFA	↑Villus height, ↓crypt depth, ↓intraepithelial lymphocytes	Weaned pigs	[51]
	Affected gastric microbial ecology, altered intestinal SCFA concentrations	Weaned pigs	[55]
	↓*Salmonella* typhimurium	*in vitro* simulation of porcine cecum	[56]
n-3 PUFA	When fed to sow during gestation and lactation periods, enriches piglet tissues and ↑intestinal structure	Piglets	[65–71]
	↓Sensitivity of epithelial barrier to mast cell degranulation	Piglets	[65]
	↑Glucose absorption, ↑protein expression of glucose transporters	Newly weaned pigs	[67, 69]
	↑Intestinal morphology and barrier function, ↓TLR4 and NOD2 signaling	LPS-challenged piglets	[2]
	↑Transepithelial electrical resistance	Suckling pigs after ischemic injury	[74]
	↓Serum endotoxin concentration, ↓*Ex vivo* mucosal to serosal endotoxin transport	Growing pigs	[75]
ARA	↓Histological lesions, ↑transepithelial resistance recovery, ↓ mucosal-to-serosal flux	Suckling pigs after ischemic injury	[74]
CLA	↓Enteric damage and clinical signs, ↑PPARγ and PGC1α, ↓TNF-α	Pig model of DSS-induced colitis	[81]
	↓Mucosal damage and inflammation, ↑PPARγ, ↓IFNγ	Pig model of bacterial-induced colitis	[11, 82]
	↓Intestinal inflammation, ↑serum IgG and IgA	ETEC-challenged piglets	[83]

*DSS* dextran sodium sulfate, *EGFR* epidermal growth factor receptor, *ETEC* enterotoxigenic *Escherichia coli*, *GI* gastrointestinal, *IFN* Interferon, *MCFA* medium-chain fatty acid, *NF-κB* nuclear factor-κB, *NOD2* nucleotide binding oligomerization domain protein 2, *PGC1α* PPAR γ-coactivator-1α, *PPARγ* proliferator-activated receptor-γ, *PUFA* polyunsaturated fatty acid, *SCFA* short-chain fatty acid, *TLR4* toll-like receptor 4, *TNF* tumor necrosis factor, *TPN* total parenteral nutrition

### SCFA

Fatty acids with a chain of less than six carbon atoms are called short-chain fatty acids (SCFA), which include acetate, propionate and butyrate. The SCFA are primarily produced by microbial fermentation of diet-resistant carbohydrates and fiber in the colon, particularly butyrate in the hindgut [35]. They are the major fuels of colonocytes and provide 60–70 % of energy requirement for colonocytes [9]. Both weaning and growing pigs have a great capacity to absorb and metabolize SCFA from the hindgut. A reduced capacity of intestinal mucosa to oxidize butyrate has been implicated in the pathogenesis of ulcerative colitis [36]. Thus, SCFA are essential for maintaining the normal metabolism of colon mucosa, regulating colonocyte growth and proliferation [10]. The beneficial effect of SCFA is not restricted to the colon, and SCFA also stimulates cell proliferation and growth of small intestine. This effect on distant mucosa is likely mediated by a systemic mediatory mechanism [37].

SCFA have been shown to play an important role on improving intestinal heath and limiting intestinal inflammation in pigs. Research in neonatal piglets has demonstrated that total parenteral nutrition (TPN) impairs gut barrier function and induces small intestine atrophy [38, 39]. However, compared to control TPN, supplementation of TPN with butyrate prevents TPN-associated small intestine mucosal atrophy and augments structural indices of GI adaptation in neonatal piglets after 80 % jejunoileal resection surgery [40]. The beneficial effect of butyrate is closely correlated with increasing proliferation and decreasing apoptosis of enterocytes [40, 41]. In addition, Fang et al. [42] reported that dietary supplementation of sodium butyrate (1 g/kg feed) significantly decreased diarrhoea incidence of weaned piglets, and enhanced the immune function by increasing the serum IgG concentration and IgA^+^ cell count in jejunum, and thus reduced the adverse effects of weaning stress and maintained the integrity of intestinal mucosa. Similarly, the earlier work in our laboratory showed that 0.5 % tributyrin improved intestinal morphology and disaccharidase activity in newly weaned pigs [43]. Moreover, our recent work also demonstrated that dietary supplementation with 0.1 % tributyrin alleviated intestinal injury by inhibiting apoptosis, promoting tight-junction formation and activating epidermal growth factor receptor signaling in a piglet colitis model induced by intrarectal administration of acetic acid [44]. Using a porcine IPEC J2 cell model, Ma et al. [45] also found that butyrate promoted the recovering of intestinal wound healing through enhanced mRNA expression of the intestinal mucosal tight junction proteins. Furthermore, Wen et al. [46] reported that sodium butyrate (1 g/kg feed) improved intestinal morphology, reduced the total viable counts of proximal colon *Clostridium* and *Escherichia coli*, decreased TNF-α and IL-6 levels in the serum and DNA-binding activity of the intestinal nuclear factor-κB in weaned piglets. Le Gall et al. [47] reported that sodium butyrate (3 g/kg dry matter intake) supplementation before weaning stimulated efficiently body growth and feed intake after weaning, by reducing gastric emptying and intestinal mucosa weight and by increasing feed digestibility. Zeng et al. [48] showed that short-chain fatty acids and their analogs induced porcine host defense peptide gene expression in IPEC-J2 intestinal epithelial cells. Taken together, dietary supplementation of butyrate to promote pig intestinal health and attenuate intestinal inflammation is a promising means.

### MCFA

Fatty acids with aliphatic tails of six to twelve carbon atoms are called medium-chain fatty acids (MCFA), which occur naturally as medium-chain triglycerides (MCT) in milk fat and various feed materials, especially coconut, palm oils and Cuphea seed oils [10]. Both MCFA and MCT have specific nutritional and metabolic effects, including rapid digestion, passive absorption and obligatory oxidation, making them particularly interesting for the nutrition of young animals [49]. MCFA can be utilized directly by the enterocytes for energy production and thereby help to support the integrity of the intestine in young piglets [50]. For example, Dierick et al. [51] reported that feeding of MCFA to weaning pigs influenced the intestinal morphology, resulting in a significant increase in the length of the villi in the small intestine combined with a lower crypt depth and a lower number of intraepithelial lymphocytes.

MCFA or MCT have been suggested to improve gut health under inflammatory conditions. However, the evidence from the pig is lacking. Bertevello et al. [52] reported that partial replacement of n-6 fatty acids with MCT improved colon cytokine response and damage in experimental colitis of rats. Papada et al. [53] found that the MCT-rich diet decreased IL-6, IL-8 and intercellular adhesion molecule-1 (ICAM-1) levels and glutathione S-transferase (GST) activity, thus exerted an anti-inflammatory effects in TNBS colitis of rat. In addition, rats fed MCT showed a significant decrease in the expression of proinflammatory cytokines and chemokines (TNF-α, IL-18, macrophage inflammatory protein-2 and monocyte chemoattractant protein-1) in the ileum and Peyer’s patches in a sepsis model of rat [54]. Based on these findings in rats, supplementing MCT or MCFA to attenuate pig intestinal inflammation might be a promising means. However, this needs to be further investigated in pigs.

Additionally, MCFA or MCT have been shown to have antimicrobial and antiviral activity in gastric lining and small intestine of pigs. Zentek et al. [55] reported that low dietary MCFA supplementation affected gastric microbial ecology, decreased propionic, butyric and valeric acid concentrations, and increased acetic acid concentration in the small intestine of weanling piglets. In addition, Messens et al. [56] found MCFA inhibited *Salmonella* typhimurium in an *in vitro* simulation of the porcine cecum. MCFA are mainly considered to be anionic surfactants, which, as a result of this property, have antibacterial effects [57]. Membrane destabilization by the incorporation of MCFA into the bacterial cell wall and cytoplasmic membrane, as well as the inhibition of bacterial lipases, which are necessary for the colonization of the skin and the intestinal mucosa, may be the cardinal mechanisms [58].

### PUFA

Essential fatty acids (EFA) are fatty acids that cannot be synthesized endogenously by animals; therefore, they must be provided exogenously from dietary sources [59]. There are two families of EFA: n-6 (or ω-6) and n-3 (or ω-3). Linoleic acid (LA; C18:2n-6) and α-linolenic acid (ALA; C18:3n-3) are the parent compounds of the n-6 and n-3 families, respectively [60]. Many plant oils, including corn, sunflower and soybean oils, are rich sources of n-6 fatty acids, mainly as LA, but linseed (flaxseed or flax) is rich in ALA. In pigs, dietary ALA and LA can be metabolized to long chain PUFA such as eicosapentaenoic acid (EPA; C20:5n-3), docosahexaenoic acid (DHA; C22:6n-3), and arachidonic acid (ARA; C20:4n-6). However, this conversion efficiency is limited due to low desaturase activity [61, 62]. For pigs, commonly available dietary sources of EPA and DHA are fish oil and algal n-3 PUFA.

Long chain PUFA play an important role on normal growth and development of pigs. Nowadays, there has been great interest in how long chain PUFA affect gut health. Modification of dietary PUFA intake substantially influences membrane structure through incorporation into cellular membrane phospholipids in many tissues including the intestine [63]. The changes in cellular membrane phospholipids results in alterations in eicosanoid synthesis, membrane fluidity, signal transduction, intraluminal bacteria, and gene expression, and thus affect cellular functions including intestinal function [64].

### n-3 PUFA

Modification of n-3 PUFA concentrations in maternal plasma, milk, and reproductive organs has been demonstrated in pig studies in which the sows were supplemented with n-3 PUFA during the gestation and lactation periods [65–68]. In addition to changes in maternal fatty acid composition in these studies, modification of fatty acid composition, structure, and physiology in intestinal tissues of the newborn and weaning pigs were observed [65, 67, 69–71]. For example, Boudry et al. [65] demonstrated n-3 PUFA supplementation in the maternal diet during gestation and lactation increased n-3 PUFA levels in maternal red blood cells and piglet ileum at birth, and on 7 and 28 days after birth. Moreover, maternal n-3 PUFA supplementation decreased villus height and crypt cell depth and the sensitivity of the epithelial barrier to mast cell degranulation of piglet ileam compared with lard-fed sows [65]. In addition, Gabler et al. [67, 69] also found that *in utero* and postnatal suckling exposure to n-3 PUFA enhanced intestinal glucose absorption in newly weaned pigs via increased protein expressions of glucose transporter 2 and sodium-dependent glucose transporter 1 potentially via the acute activation of AMP-activated protein kinase. Moreover, De Quelen et al. [72] showed that maternal n-3 PUFA modified intestinal permeability probably via diet-induced neuroplastic changes in the intestinal nervous system of newborn pigs compared with lard-fed sows. Desaldeleer et al. [73] reported that C18:3n-3 supplementation in the maternal diet favored piglet intestinal passage of LPS and promoted intestinal anti-inflammatory response to LPS compared to a maternal C18:2n-6 diet.

Abundant literatures have demonstrated that n-3 PUFA exert beneficial effects on inflammatory bowel diseases in animal models and clinical trials [6–8]. However, relatively little attention has been given to n-3 PUFA on intestinal health of pigs under inflammatory conditions. Recently, we conducted an experiment to investigate if fish oil (rich in EPA and DHA) could alleviate *Escherichia coli* LPS-induced intestinal injury in weaned pigs. We found that, compared with corn oil, 5 % fish oil improved intestinal morphology indicated by greater villus height and villus height/crypt depth ratio, and intestinal barrier function indicated by decreased plasma diamine oxidase activity and increased mucosal diamine oxidase acitivity as well as enhanced protein expression of intestinal tight junction proteins including occludin and claudin-1 independent of LPS challenge [2]. It has been demonstrated that the beneficial roles of fish oil on intestinal injury are correlated with inhibition of toll-like receptor 4 and nucleotide binding oligomerization domain protein 2 signaling pathways and downregulation of pro-inflammatory mediators such as TNF-α and prostaglandin E_2_ [2]. In addition, Jacobi et al. [74] showed that dietary supplementation of 5 % EPA enhanced transepithelial electrical resistance in ischemic-injured ileum of 1-day old suckling pigs. Mani et al. [75] reported that n-3 PUFA reduced postprandial serum endotoxin concentration and *Ex vivo* mucosal to serosal endotoxin transport permeability in growing pigs compared with no oil control.

### ARA

Generally, it is thought that n-3 PUFA is “good” as anti-inflammatory and n-6 PUFA is “bad” as proinflammatory in the literatures. However, some studies have shown that n-6 PUFA, especially ARA as well as its metabolites, facilitate recovery of damaged intestinal mucosa. Ruthig and Meckling-Gill. [76] reported that ARA significantly enhanced cellular migration of the rat intestinal epithelial cell line, IEC-6 after razor wounding, a model of intestinal restitution. Further examination revealed that greater prostaglandin E_2_ production in ARA-supplemented cultures, and ARA-stimulated migration being attenuated by cyclooxygenase 2 inhibitors [77], which demonstrates that ARA enrichment in intestinal cells enhances prostaglandin E_2_ production and stimulates restitution. These results in rats indicate an important role of ARA in stimulating recovery of damaged intestinal mucosa. In addition, the studies in pigs have also shown that prostanoids stimulate rapid recovery of barrier function as indicated by elevated transepithelial resistance, and restore baseline levels of permeability after ischemic injury [78, 79]. Recently, Jacobi et al. [74] also showed that 5 % ARA attenuated histological lesions, increased transepithelial resistance recovery, and inhibited mucosal-to-serosal flux of ^3^H-mannitol and ^14^C-inulin after ileal ischemia in 1-day old suckling pigs.

### CLA

CLA are a group of positional and geometric isomers of linoleic acid. CLA are characterized by the presence of conjugated dienes and differ in both the position and the stereochemistry of their double bonds [10]. Naturally occurring CLA are produced mainly from bacterial isomerisation and biohydrogenation of PUFA in the rumen and the desaturation of trans-fatty acids in mammary gland and adipose tissue [10]. In synthetic CLA preparations, the cis-9, trans-11 and trans-10, cis-12 isomers predominate, often in 1:1 ratios [80]. These two isomers, which have been used in experimental studies as a mixture, represent the most widely investigated CLA isomers.

Conjugated linoleic acids have been demonstrated to exert beneficial effects in several pig colitis models. Bassaganya-Riera and Hontecillas [81] reported that 1.33 % CLA delayed the onset of enteric damage and attenuated the clinical signs in a pig model of dextran sodium sulfate-induced colitis compared to soybean oil, which is correlated with the induction of colonic proliferator-activated receptor-γ (PPARγ) and its responsive gene PPAR γ-coactivator-1α (PGC1α) and downregulation of TNF-α [81]. Bassaganya-Riera et al. [82] also found that CLA attenuated intestinal inflammation in a bacterial-induced model of colitis, which is also associated with the induction of PPARγ expression and reduction of IFNγ expression. In addition, Hontecillas et al. [11] reported that supplementation of 1.33 % CLA in the diet before the induction of colitis decreased mucosal damage, maintained cytokine profiles and lymphocyte subset distributions, and enhanced colonic expression of PPARγ in the bacterial-induced colitis model of pigs compared to soybean oil. Moreover, Patterson et al. [83] found that piglets weaned from 2 % CLA-supplemented sows showed reduced intestinal inflammation and increased serum IgG and IgA compared to piglets weaned from control sows after enterotoxigenic *Escherichia coli* (ETEC) challenge. Although there were no obvious additional health effects observed when CLA was provided in nursery diet, supplementing sow rations with 2 % CLA from mid-gestation through weaning appeared to have immune-stimulating carry-over effects post weaning [83]. Thus, supplementing sow rations with CLA is a practical strategy for enhancing overall gut health of nursery piglets [83].

## Conclusions

Activation of GI immune system associated with “normal” commensal microbiota, activation of overt inflammatory responses associated with enteric pathogen exposures as well as inflammation induced by stresses such as weaning, has significant implications on nutrient assimilation and utilization by the pig. Major physiological changes in intestine include impaired intestinal morphology, decreased digestive enzyme activity, and impaired barrier functions such as mucin secretion and tight-junction proteins. Thus, it is important to develop nutritional strategies to maintain or improve intestinal integrity and function under inflammatory conditions. Recent studies in pigs indicate that specific fatty acid including short chain and medium chain fatty acids and long chain PUFA play potential therapeutic roles in intestinal inflammation in pigs. Results of these new lines of work indicate trophic and cytoprotective effects of fatty acids on intestinal integrity in pigs. Utilization of these nutritional strategies may offer considerable opportunity to improve pig health and the efficiency of nutrient use for human food production.

## Abbreviations

ALA: α-linolenic acid
APN: Aminopeptidase N
ARA: Arachidonic acid
CLA: Conjugated linoleic acid
DHA: Docosahexaenoic acid
EFA: Essential fatty acid
EPA: Eicosapentaenoic acid
ETEC: Enterotoxigenic *Escherichia coli*
GGT: Gamma-glutamyl transpeptidase
GI: Gastrointestinal
GST: Glutathione S-transferase
ICAM-1: Intercellular adhesion molecule-1
IFN: Interferon
IL: Interleukin
LA: Linoleic acid
LPH: Lactase phloryzin hydrolase
MCFA: Medium-chain fatty acid
MCT: Medium-chain triglyceride
PGC1α: PPAR γ-coactivator-1α
PPARγ: Proliferator-activated receptor-γ
PUFA: Polyunsaturated fatty acid
SCFA: Short-chain fatty acid
TNF: Tumor necrosis factor
TPN: Total parenteral nutrition
